# The impact of genetic counseling on parental risk perception and prevention strategy utilization for type 2 diabetes in at-risk children

**DOI:** 10.1007/s12687-025-00841-z

**Published:** 2025-11-17

**Authors:** Jessica Denton, Madison Nee, Gerald McGwin, Andrea Frickman

**Affiliations:** 1 School of Health Professions, University of Alabama at Birmingham, Birmingham, AL USA; 2 Center for Clinical and Translational Sciences, University of Alabama at Birmingham, Birmingham, AL USA

**Keywords:** Risk perception, Family history, Type 2 diabetes, Prevention strategies, Genetic counseling, Children, Diverse population

## Abstract

Family history is a strong risk factor for type 2 diabetes (T2D), but few studies have evaluated if parents with T2D understand their child’s risk and appropriate prevention strategies. Genetic counseling is an effective intervention to communicate risk for multifactorial disease and provide counseling on management and prevention. This prospective, cross-sectional feasibility study evaluates the impact of a genetic counseling intervention on parental risk perception towards T2D, knowledge of prevention strategies, and implementation of prevention strategies for their at-risk children. Thirty-seven parents with T2D and children between 2 and 11 years old were randomized into an intervention (*n* = 18) or control (*n* = 19) group. The intervention group received a structured genetic counseling intervention, which included an educational video, personalized risk assessment, and discussion of prevention strategies. Surveys measuring risk perception, knowledge of prevention strategies, patient empowerment, and health behaviors were administered pre-intervention and at one-month post-intervention for both groups. At one-month post-intervention, the intervention group demonstrated a statistically significant increase in risk knowledge (*p* = 0.0185) and higher engagement in monitoring child-specific dietary behaviors (*p* = 0.0334) compared to the control group while changes in overall risk perception, knowledge of prevention strategies, and family dietary habits were not significant. This study demonstrates that genetic counseling could be an effective method to increase parental risk knowledge and engagement in child-specific preventive behaviors for T2D. Findings from this study’s unique population of diverse parents with young, healthy children who have yet to develop modifiable risk factors for T2D could provide insight for developing targeted prevention programs.

**Supplementary Information:**

The online version contains supplementary material available at 10.1007/s12687-025-00841-z.

## Introduction

Type 2 diabetes (T2D) is becoming one of the most common chronic conditions worldwide among children, and its prevalence is estimated to increase 700% in the United States by 2060 (Tonnies et al., 2023). Youth-onset T2D is characterized by pronounced insulin resistance, increased insulin secretion, and rapid pancreatic beta-cell decline, resulting in complications such as cardiovascular disease, neuropathy, retinopathy, and nephropathy, which are more severe and arise more quickly than in adult-onset cases (Song and Hardisty 2009; Hitt et al. 2023). Given the alarming trends in incidence and the morbidity associated with the disease, it is important to understand risk factors for T2D in children to develop appropriate prevention strategies.

Previous studies have identified two main types of risk factors that contribute to youth-onset T2D: modifiable factors, such as diet and physical activity, and non-modifiable factors, including ethnicity and family history (Arslanian et al. 2018). When considering ethnicity, the prevalence of T2D is disproportionately higher among youth from non-White backgrounds. In 2017, the prevalence of T2D among adolescents of non-Hispanic Black ethnicity was more than 4 times higher, and nearly 2.5 times higher among adolescents of Hispanic ethnicity compared to adolescents of non-Hispanic White ethnicity (Lawrence et al. 2021). Family history has also consistently emerged as one of the strongest non-modifiable risk factors for T2D. Studies estimate that 74–100% of youth diagnosed with T2D have an affected first- or second-degree relative (Strati et al. 2024), and up to 90% report a positive family history (Goyal and Vanita 2025). Lifetime risk estimates suggest a 40% risk for a child with one affected parent and up to 70% if both are affected with T2D (Goyal and Vanita 2025). Together, these findings highlight the strong association of family history and ethnicity on youth-onset T2D, reinforcing the need for early identification and targeted prevention efforts for at-risk children.

Child-focused interventions have commonly used family-based designs, which consistently show parental engagement in their children’s diet and physical activity enhances intervention effectiveness and sustainability (Kurtzhals et al. 2024). The EPIC Kids Study, a family-centered YMCA-based lifestyle intervention, observed an increase in children’s physical activity and a reduction in waist circumference when parents were actively involved (Hingle et al. 2015). Similarly, a systematic review of family-adapted diabetes prevention programs found that incorporating family members led to improved health behaviors, such as increased physical activity, healthier eating patterns, and greater adherence to intervention goals, compared to youth-only programs (Andreae et al. 2024). While these studies demonstrate the effectiveness of family-based strategies in improving health behaviors, they do not examine the psychological or cognitive factors, such as risk perception and knowledge of prevention strategies, that may influence whether families engage with and sustain these changes.

As outlined in the Health Belief Model, risk perception, an individual’s subjective assessment of disease likelihood, is a key driver of preventive behavior (Brewer et al. 2007). For multifactorial conditions such as T2D, cognitive, psychosocial, and familial influences all shape how individuals interpret their risk. Because of these influences, studies have found that variation can exist between individual risk perception and clinical risk assessment. For example, Daack-Hirsch et al. (2020) found that 23% of adults with a family history of T2D accurately perceived themselves to be at increased risk. Airikkala et al. (2023) noted that while family history increased perceived risk across samples, interpretations varied, with some individuals viewing heredity as motivating and others as making diabetes inevitable. Khosrovaneh et al. (2025) further reported that while 81% of adults at elevated risk recognized diabetes as a serious health condition, only 46% perceived it as preventable and 39% felt confident in their ability to reduce their risk.

When considering parental risk perception, additional considerations are introduced, such as protective instincts and emotional responses. One factor influencing this is optimistic bias; Wright et al. (2016) observed that parents rated their own child’s likelihood of developing T2D at 12.1%, compared to 25.8% for same-age peers, even while acknowledging obesity-related conditions such as cardiovascular disease as potential outcomes. At the same time, parents have expressed readiness to engage in prevention if given appropriate support. In a Malaysian cohort, Badlishah-Sham et al. (2020) found that 62% of parents with T2D were interested in training to help them talk with their children about diabetes prevention, suggesting strong potential for family-based education to promote risk awareness and implementation of prevention strategies.

Genetic counselors are well-positioned to fill this educational role. As health professionals trained to conduct risk assessments and communicate risk and prevention strategies for hereditary conditions, they possess skills that translate well to the context of T2D (Resta 2019). A recent practice guideline from the National Society of Genetic Counselors emphasized the effectiveness of genetic counseling for multifactorial conditions, including T2D (Maloney et al. 2023). The guideline highlighted the role of genetic counselors in helping patients understand complex genetic risks and apply that knowledge to make informed lifestyle changes for prevention and management. For families with a history of T2D, genetic counseling interventions may enhance understanding of risk and awareness of prevention strategies. Prior studies, such as Wu et al. (2017), have shown that genetic counseling interventions can influence adults’ perception of diabetes risk and health behaviors, but these interventions have largely focused on adults assessing their own risk. Similar approaches have not been applied to parents evaluating their children’s risk, particularly in diverse populations.

This study addresses gaps in current literature by evaluating a structured genetic counseling intervention for parents with T2D. Specifically, it examines how counseling affects parental perception of their child’s risk, understanding of prevention strategies for their children, and adoption of lifestyle changes to reduce the likelihood of youth-onset T2D. By centering on the perspectives of parents and engaging families from diverse backgrounds, this work extends current prevention research and demonstrates a novel application of genetic counseling in multifactorial disease prevention.

## Methods

### Study design

This prospective, cross-sectional feasibility study was conducted between March 2021 and January 2022. The study received approval from The University of Alabama at Birmingham (UAB) Institutional Review Board (IRB-300006670-10).

### Participants and recruitment

Participants were recruited from multiple sources, including the University of Alabama at Birmingham (UAB) Endocrinology Clinic, an obstetrics clinic for diabetes patients, and individuals previously screened for the Medical Optimization and Management of Pregnancies with Overt Type 2 Diabetes (MOMPOD) study at UAB. Eligible participants were adults diagnosed with T2D, who were the biological parent of a child aged 2–11 years, with whom the child primarily resided. Exclusion criteria included a diagnosis of type 1 diabetes or T2D in the child or children with medical conditions that affect diet or metabolism.

**Fig. 1:**
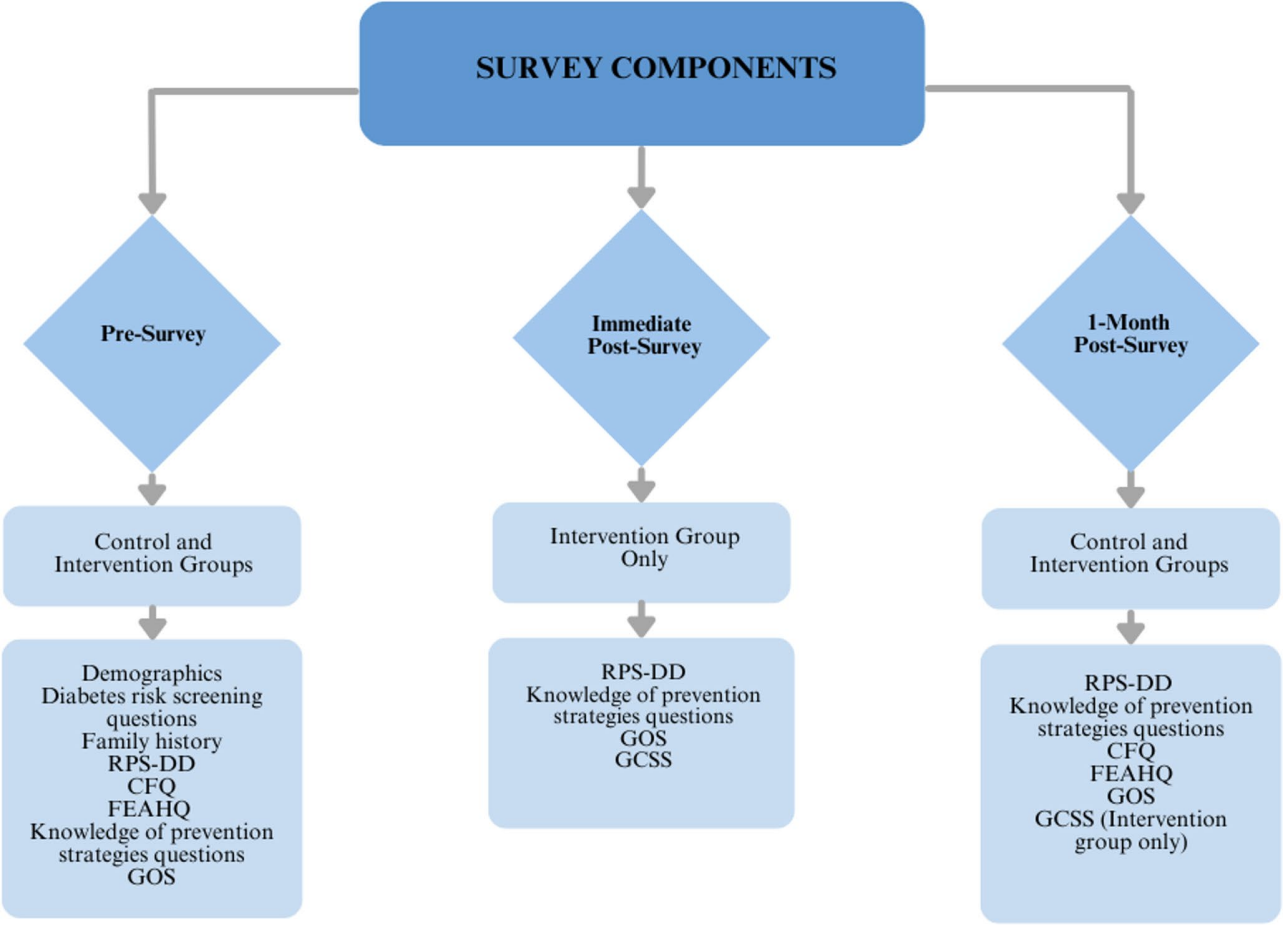
Survey components across study time points and participant groups

Participants were recruited both in-person and virtually. In-person recruitment took place at the UAB Obstetrical Complications Clinic (OBCC) Diabetes Clinic, where potential participants were screened based on electronic medical record review for T2D diagnosis and having a child in the specified age range. Eligible participants were approached, then asked additional screening questions related to the inclusion and exclusion criteria. If patients met the criteria, they were provided basic information about the study, and if interested, they were fully consented and began the study during their clinic visit. Virtual recruitment involved sending patient portal messages to endocrinology patients who were screened via their electronic medical record for a T2D diagnosis. These individuals were sent a link to complete an eligibility survey that contained the inclusion and exclusion criteria via the patient portal. If they met criteria, they were directed to an online consent form and contacted by study personnel once the consent form was completed. Individuals from the MOMPOD study who agreed to be re-contacted for future research studies were screened via the medical record for eligibility. In a similar process to the endocrinology patients, they were either sent a portal message or called and screened over the phone if they did not have access to the patient portal. If screened over the phone, they were emailed the link to the consent form. Patients who consented to participate were randomized into the intervention group or the control group via a block randomization method. Participants who were recruited in the OBCC clinic conducted all parts of the study in person. Participants who were recruited through endocrinology and MOMPOD completed the study virtually using HIPAA-secure Zoom. All participants completed a pre-survey and a survey 1-month after the pre-survey. Participants randomized to the intervention group completed the pre-survey, underwent the genetic counseling intervention, then completed a survey directly after the intervention in addition to the survey 1-month later. If participants had more than one child between 2 and 11 years old, they were asked to complete the survey regarding their youngest child within the specified age range. Elements of each survey across timepoints are presented in Fig. 1.

The surveys for this study were administered through REDCap, a secure online survey platform that facilitated both in-person and virtual delivery options (Harris et al. 2019). Participants completed the pre-survey either on paper in the OBCC clinic or through a secure link provided during a HIPAA-compliant Zoom video call. Participants received a gift card for ten dollars after completion of the 1-month post-survey.

### Genetic counseling intervention

Participants in the intervention group took part in a structured genetic counseling session, which began with a 10-minute educational video titled “Type 2 Diabetes in Children: Risks and Prevention.” This video was developed by a certified genetic counselor using Microsoft PowerPoint with narration. The video covered several key topics: the incidence of childhood T2D, risk factors for developing childhood T2D, the influence of family history on T2D risk, the roles of genetics and environment in T2D development, and prevention strategies for reducing T2D risk in children from the Centers for Disease Control and Prevention. Following the video, the genetic counseling session was guided by a formal session outline and included four main components:

- *Video Debrief*: Participants were given an opportunity to ask questions and discuss the information presented in the educational video.
- *Risk Communication:* The genetic counselor (GC) used information from the participant’s family history and demographic data collected in the pre-survey to provide a personalized risk assessment for their child. This personalized approach aimed to help participants understand the specific factors that may influence their child’s risk such as number of affected family members and the child’s health status.
- *Prevention Strategy Discussion:* The GC and participant evaluated which prevention strategies could be realistically implemented within the family’s lifestyle while taking into consideration current health habits of their child and family. Participants were encouraged to choose 2-3 strategies that were new for them and that were feasible to implement within a one-month time frame.
- *Session Summary:* The session concluded with a summary of the child’s T2D risk, confirmation of the prevention strategies the participant planned to implement over the next month, a discussion of the next steps for the study, and an opportunity for the participant to ask any remaining questions.

All participants received a link to the video and additional educational materials related to prevention strategies for children at the conclusion of the study regardless of study group.

### Survey measures

#### Risk perception

Risk perception was measured using Risk Perception for the Development of Diabetes (RPS-DD) survey, a validated survey that assessed the participants’ perception of their child’s risk of developing diabetes (Elizabeth et al. 2003). Questions in the personal control, worry, and optimistic bias sections were slightly modified to focus participants on their child’s risk rather than their own. For example, ‘my child’s’ was used instead of ‘my’ in questions such as “I feel that I have little control over risks to *my child’s* health.” In addition, participants were asked to answer questions in the personal disease risk and comparative environmental risk sections by thinking about their child rather than themselves, though no wording of questions was modified in these sections. The responses were scored using the scoring guide developed by the creators of the survey to calculate subscale scores and a composite risk perception score (Rochefort et al. 2020). *Knowledge of prevention strategies*.

A 15-question survey related to the information provided in the educational T2D video was developed by the authors to assess the intervention’s impact on participant knowledge of prevention strategies for their children. Responses were scored by summing the Likert scale answers (0 = I don’t know; 5 = definitely yes, with definitely yes being the correct answer for all questions). Higher scores demonstrated higher knowledge of prevention strategies.

#### Implementation of prevention strategies

To assess health behaviors of the participants and their families, two validated questionnaires were used— the Child Feeding Questionnaire (CFQ) and the Family Eating and Activity Habits Questionnaire (FEAHQ). The CFQ was used to assess parental beliefs, attitudes, and practices in relation to child feeding (Birch et al. 2001). Only certain subscales of the CFQ were included in the survey based on their relevance to the prevention strategies discussed in the video. These subscales included perceived responsibility, restriction, pressure to eat, and monitoring. Subscales were scored according to the scale creators by calculating the mean score for each item on a Likert scale (1 = never, disagree; 5 = always, agree). The total score was also calculated. The FEAHQ was used to assess the eating and activity habits of family members (Golan and Weizman 1998). The following subscales were included in the survey based on their relevance to the prevention strategies: stimulus exposure, eating related to hunger, and eating style. The total score was also calculated. Responses were scored concordantly with the scoring guide developed by the FEAHQ creators.

#### Patient empowerment

The Genomics Outcome Scale (GOS) was used to evaluate patient empowerment (Grant et al. 2019). The Genetic Counseling Satisfaction Scale (GCSS) was included for the intervention group only to measure satisfaction with the genetic counseling received (Tercyak et al. 2001).

### Statistical analyses

Paired t-tests were used to determine if there were significant within-group differences between two time points. To determine the immediate effect of the intervention, the intervention group scores from the RPS-DD, knowledge of prevention strategies questions, and GOS were compared between pre-survey and immediate post-survey. As the CFQ and FEAHQ were only administered at pre-survey and 1-month post-survey, within-group differences for these surveys were also determined using paired t-tests for both the intervention group and the control group.

One-way repeated measures analysis of variance (ANOVA) was used to determine significant within-group differences when data was collected at three time points. The differences in RPS-DD, knowledge of prevention strategies, and GOS scores across the pre-survey, immediate post-survey, and 1-month post-survey time points were calculated using this method for the intervention group only.

Two-way mixed analysis of covariance (ANCOVA) was used to evaluate between-group differences in the RPS-DD, knowledge of prevention strategies, CFQ, FEAHQ, and GOS scores between the intervention and control groups at 1-month post-survey. Pre-survey scores were included as a covariate to control for any pre-survey score variability that could contribute to between-group differences. No significant differences were noted between the demographics of the control group and intervention group, so these variables were not included in the ANCOVAs.

*P*-values of ≤ 0.05 (two-sided) were considered statistically significant. All statistical analyses were conducted using SAS, version 9.4.

## Results

### Participants

A total of 37 individuals participated in the study with 19 randomized to the control group and 18 to the intervention group. The mean age of participants was 32 years old, and most participants were female (*n* = 35, 94.6%), identified as Black (*n* = 28, 75.7%) and had attained at or above a high school education (*n* = 33, 89.2%) (Table 1). Characteristics of the participant’s child was also collected (Table 2). The majority of children were female (*n* = 21, 56.76%), with a mean age of 6 years old, and the majority of the participants felt like their children had healthy diets (*n* = 27, 73.97%) and got 60 min of exercise each day (*n* = 31, 83.88%).

**Table 1:** Participant demographics *n* = 37

Age	
Mean Current Age (range)	32 years (23–48)
**Sex Assigned at Birth** *n* (%)	
Female	35 (94.59%)
Male	2 (5.41%)
**Race/Ethnicity** *n* (%)	
Black	28 (75.68%)
White	5 (13.51%)
Hispanic	3 (8.11%)
Native American	1 (2.70%)
**Highest Education Level** *n* (%)	
Some High School	4 (10.81%)
High School/GED	14 (37.84%)
Some College	4 (10.81%)
Associate’s Degree	9 (24.32%)
Bachelor’s Degree	3 (8.11%)
Graduate Degree	3 (8.11%)

**Table 2:** Child characteristics

Age		
Mean age (range)	6 years (2–11)	
**Sex Assigned at Birth**	***n*** (%)	
Female		21 (56.76%)
Male		16 (43.24%)
**Parent’s Perspective**		***n*** (%)
Number of children that have been told by provider they are overweight		5 (13.51%)
Number of children that get 60 min of exercise a day		31 (83.88%)
Number of children that have a healthy diet		27 (73.97%)
**Child’s Family History of T2D**		
Mean number of people in the family with type 2 diabetes including the participant (range)		2.35 (1–6)

### Risk perception

#### Intervention group

The average risk perception scores (composite score from the RPS-DD) decreased among the intervention group participants from the pre-survey to the immediate post-survey, but this change was not statistically significant (Table 3). The average scores for the optimistic bias, personal control, and comparative environment subscales of the RPS-DD also decreased, but not significantly. The average scores for the worry and risk knowledge subscales of the RPS-DD increased, but not significantly.

**Table 3:** Pre-Survey to immediate Post-Survey comparisons of risk Perception, knowledge of prevention Strategies, and the genomics outcome scale among the intervention group

Measure	Pre-Survey Mean (SD)	Immediate Post-Survey Mean (SD)	Mean Difference (SD)	*p*-value
*Risk perception (RPS-DD)* *n* = 18				
Worry	2.42 (0.48)	2.69 (0.64)	−0.3056 (0.94)	0.1864
Optimistic Bias	2.53 (0.66)	2.44 (0.78)	0.0556 (0.84)	0.782
Personal Control	3.34 (0.92)	3.22 (0.52)	0.1667 (0.53)	0.1986
Comparative Environment	1.37 (0.52)	1.33 (0.51)	0.0617 (0.16)	0.1161
Risk Knowledge	5.95 (2.27)	6.56 (1.69)	−0.6316 (1.57)	0.0967
Composite Score	1.57 (0.29)	1.55 (0.31)	0.0139 (0.1547)	0.7079
*Knowledge of Prevention Strategies* *n* = 16				
Total Score	49.64 (11.41)	53.89 (13.95)	−4.81 (12.7)	0.1497
*Genomics Outcome Scale (GOS)* *n* = 16				
Total Score	24.64 (3.10)	25.75 (2.59)	−1.111 (4.07)	0.0049*

An * indicates results that were statistically significant. Paired t-tests were used to determine differences between pre-survey and immediate post-survey scores

When comparing RPS-DD scores of the intervention group across the pre-survey, immediate post-survey, and 1-month post-survey, the only significant difference was noted within the risk knowledge subscale, where scores increased consistently across each survey and achieved statistical significance (*p* = 0.0310) (Table 4). Composite RPS-DD scores, along with worry, optimistic bias, and comparative environment subscale scores decreased from the immediate post-survey to 1-month post-survey.

**Table 4:** Comparison of changes within the intervention group across Pre-Survey, immediate post-Survey, and 1-Month post survey

Measure	Pre-Survey LS Mean (SD)	Immediate Post-Survey LS Mean (SD)	1-Month Post-Survey LS Mean (SD)	*p*-value
*Risk Perception (RPS-DD)*				
Worry	2.39 (0.18)	2.69 (0.18)	2.44 (0.19)	0.2760
Optimistic Bias	2.50 (0.16)	2.44 (0.16)	2.31 (0.17)	0.5597
Personal Control	3.39 (0.12)	3.22 (0.12)	3.45 (0.12)	0.2854
Comparative Environment	1.30 (0.12)	1.33 (0.12)	1.03 (0.12)	0.4788
Risk Knowledge	5.89 (0.55)	6.56 (0.55)	7.20 (0.55)	0.0310*
Composite Score	1.58 (0.07)	1.56 (0.07)	1.55 (0.07)	0.9128
*Knowledge of Prevention Strategies*				
Total Score	49.74 (2.61)	59.39 (2.6)	54.19 (2.61)	0.0020*
*Genomics Outcome Scale (GOS)*				
Total Score	23.13 (0.71)	24.72 (0.71)	25.81 (0.74)	0.0378*

An * indicates results that were statistically significant. One-way repeated measures analysis of variance (ANOVA) was used to determine differences across the survey time points

RPS-DD: Pre-survey *n*=18; immediate post-survey *n*=18; 1-month post survey *n*=17

Knowledge of prevention strategies: Pre-survey *n*=17; immediate post-survey *n*=18; 1-month post survey *n*=17

GOS: Pre-survey *n*=18; immediate post-survey *n*=18; 1-month post survey *n*=16

#### Intervention versus control group

When comparing RPS-DD scores between the control and intervention groups at the 1-month post-survey, the risk knowledge subscale scores were significantly higher in the intervention group than the control group (*p* = 0.0185) (Table 5). Worry, optimistic bias, and comparative environment subscale scores were higher among the control group than the intervention group, whereas the intervention group had higher personal control subscale scores, though these results were not statistically significant.

**Table 5:** Risk Perception, knowledge of prevention Strategies, and genomics outcome scale comparisons among the control and intervention groups at 1-Month post survey

Measure	Control Group 1-Month Post-Survey LS Mean	Intervention Group 1-Month Post-Survey LS Mean	Significance between groups (*p*)
*Risk Perception (RPS-DD)*			
Worry	2.97	2.47	0.0665
Optimistic Bias	2.47	2.23	0.2252
Personal Control	3.22	3.43	0.2854
Comparative Environment	1.44	1.31	0.3575
Risk Knowledge	5.02	7.16	0.0185*
Composite Score	1.67	1.59	0.4400
*Knowledge of Prevention Strategies*			
Total Score	45.24	52.91	0.0693
*Genomics Outcome Scale (GOS)*			
Total Score	23.73	25.87	0.0883

An * indicates results that were statistically significant. Two-way mixed analysis of covariance (ANCOVA) was used to evaluate differences between the intervention group and control group at one-month post-intervention. Mean pre-survey scores were used as a covariate in each ANCOVA

RPS-DD: Control group *n*=15; Intervention group *n*=17

Knowledge of prevention strategies: Control group *n*=15; Intervention group *n*=17

GOS: Control group n=15; Intervention group *n*=16

### Knowledge of prevention strategies

#### Intervention group

Knowledge of prevention strategies scores improved among the intervention group from pre-survey to immediate post-survey but did not reach statistical significance (Table 3). When comparing the changes of scores among the intervention group across the pre-survey, immediate post-purvey, and 1-month post-survey, knowledge of prevention strategies did increase significantly (*p* = 0.0020) (Table 4). Scores peaked at the immediate post-survey but declined slightly at the 1-month post-survey.

#### Intervention versus control group

The knowledge of prevention strategies scores between the control and intervention group at 1-month post-survey showed no significant differences (Table 5). However, the intervention group had higher scores than the control group.

### Implementation of prevention strategies

#### Intervention group

From pre-survey to 1-month post-survey, the intervention group’s average CFQ composite scores improved significantly (*p* = 0.0059) whereas the change in their FEAHQ scores was not significant (Table 6). The CFQ food monitoring subscale scores also significantly increased (*p* = 0.0238), and restriction subscale scores almost remained unchanged with a 0.01 decrease between pre-survey and 1-month post-survey. CFQ pressure to eat subscale scores decreased, and perceived responsibility scores increased, though not significantly. In contrast, none of the changes in the intervention group’s FEAHQ subscale scores were statistically significant, but all scores trended downward at 1-month post-survey.

**Table 6:** Pre-Survey to 1-Month Post-Survey comparisons of intervention group implementation

Measure	Pre-Survey Mean (SD)	1-Month Post-Survey Mean (SD)	Mean Difference (SD)	*p*-value
*Child Feeding Questionnaire (CFQ)* *n* = 17				
Restriction	3.64 (0.83)	3.63 (0.74)	0.04 (0.98)	0.8547
Pressure to Eat	3.12 (0.94)	2.68 (0.97)	0.47 (1.05)	0.0839
Monitoring	3.74 (1.02)	4.5 (0.73)	−0.82 (1.36)	0.0238*
Perceived Responsibility	4.7 (0.55)	4.83 (0.38)	−0.098 (0.094)	0.1631
CFQ Composite Score	13.68 (1.87)	15.03 (1.58)	−1.46 (1.86)	0.0059*
*Family Eating and Activity Habits Questionnaire (FEAHQ)* *n* = 17				
Stimulus Exposure	13.68 (4.6)	11.53 (4.93)	1.71 (4.09)	0.1047
Eating Related to Hunger	3.53 (1.87)	2.95 (1.87)	0.29 (2.02)	0.5574
Eating Style	33.11 (7.24)	28.68 (11.76)	3.65 (11.7)	0.2180
FEAHQ Composite Score	50.32 (9.5)	43.16 (14.69)	5.65 (13.3)	0.0991

An * indicates results that were statistically significant. Paired t-tests were used to determine differences between pre-survey and one-month post-survey scores

#### Intervention versus control group

When comparing implementation outcomes between the intervention and control groups at the 1-month post-survey, CFQ composite scores were significantly higher in the intervention group (*p* = 0.0334) (Table 7). However, no significant differences were observed between groups in the FEAHQ composite scores. CFQ monitoring, restriction, and perceived responsibility scores were higher in the intervention group than the control group, although not statistically significant independently.

**Table 7:** Implementation comparisons between control and intervention group at 1-month post-survey using pre-survey scores as an independent variable

Measure	Control Group 1-Month Post-Survey LS Mean	Intervention Group 1-Month Post-Survey LS Mean	Significance between groups (*p*)
*Child Feeding Questionnaire (CFQ)*			
Restriction	3.59	3.63	0.8635
Pressure to Eat	2.99	2.60	0.1579
Monitoring	4.01	4.54	0.0788
Perceived Responsibility	4.72	4.80	0.6691
Composite Score	14.10	15.15	0.0334*
*Family Eating and Activity Habits Questionnaire (FEAHQ)*			
Stimulus Exposure	12.21	12.05	0.9056
Eating Related to Hunger	3.22	3.16	0.9245
Eating Style	30.85	29.72	0.7181
Composite Score	46.39	44.84	0.6579

An * indicates results that were statistically significant. Two-way mixed analysis of covariance (ANCOVA) was used to evaluate differences between the intervention group and control group at one-month post-intervention. Mean pre-survey scores were used as a covariate in each ANCOVA

### Genomics outcome scale (GOS)

#### Intervention group

GOS scores increased significantly in the intervention group participants from pre-survey to immediate post-survey (*p* = 0.0049) (Table 3). This improvement persisted from immediate post-survey to 1-month post-survey scores, with a significant upward trend across all time points (*p* = 0.0378) (Table 4).

#### Intervention versus control group

Compared to the control group, the intervention group had higher GOS scores at the 1-month post-survey, although this difference was not statistically significant (Table 5).

### Genetic counseling satisfaction scale (GCSS)

The GCSS was administered to the intervention group only. Among the 15 responses, average satisfaction scores decreased slightly from 4.8/5 directly after the intervention to 4.71/5 on the 1-month post-survey. Due to the lack of variability in satisfaction scores, we did not investigate which demographic factors could be contributing to satisfaction.

## Discussion

This study evaluated the impact of a genetic counseling intervention on parents with type 2 diabetes (T2D) on their risk perception, knowledge of childhood T2D prevention strategies, and implementation of preventive behaviors. Findings suggest that genetic counseling can increase parental risk knowledge and improve prevention efforts related to child feeding behaviors. The effect of genetic counseling on overall risk perception and broader family health behaviors was more variable. While modest in scale, the results highlight both the potential contributions of genetic counseling to pediatric diabetes prevention and areas for intervention refinement for future research.

### Main findings

Despite increased risk knowledge, overall risk perception scores and other subscales of the RPS-DD, such as worry and optimistic bias, were not significantly different when comparing the control group to the intervention group. Moreover, the intervention group within group RPS-DD scores did not change significantly. It is possible that participants were already aware of their child’s increased risk of developing T2D prior to the genetic counseling intervention. Even though the information provided in the intervention was specific to their child, if parents already believed their child was at increased risk, a personalized risk assessment may have simply reinforced that belief without shifting their overall perception.

On the other hand, given the children in this study were young, and engaging in healthy habits, participants may not have perceived their child to be at risk due to lack of risk factors beyond family history and ethnicity, and that perception was not changed by the intervention. Prior research shows that perceptions of risk are not solely determined by knowledge but also by psychological factors such as worry, optimism, and self-efficacy (Airikkala et al. 2023; Khosrovaneh et al. 2025). All of these components were not measured in this study, and those that were (e.g., worry), may change over time as additional risk factors are acquired and children age. During the intervention, more time was spent discussing prevention strategies with participants than the factors contributing to how they perceive their child’s risk for T2D. Future studies and/or genetic counseling interventions may benefit from more time spent quantifying T2D risk perception and its influences in order to better understand parental risk perception specifically for young children with minimal to no modifiable risk factors. Future research may also aid in developing better tools to measure parental risk perception for T2D, as adapting the RPS-DD for parents to evaluate risk for their child rather than themselves may not accurately capture parental perception of pediatric risk.

The intervention’s focus on prevention strategy education and counseling could account for the statistically significant increase in knowledge of prevention strategies seen in the intervention group. Scores improved from pre-survey to immediate post-intervention and remained elevated at the one-month follow-up, though with a slight decline. As demonstrated by others, structured educational interventions can enhance diabetes-related health literacy and encourage individuals to engage in preventive behaviors (Andreae et al. 2024). (Kurtzhals et al. 2024). While knowledge retention over time suggests the intervention was effective, the slight decline at the 1-month post-survey and lack of significant difference between the intervention and control groups at 1-month indicates that continued reinforcement may be necessary for sustained knowledge maintenance.

Prevention strategy counseling for the intervention group may have also led to the statistically significant higher monitoring of child food intake, as measured by the Child Feeding Questionnaire (CFQ), compared to the control group. Increased monitoring has been associated with healthier dietary behaviors in children, which is critical for T2D prevention (Birch et al. 2001), so this is an encouraging outcome. However, while Family Eating and Activity Habits Questionnaire (FEAHQ) results suggested directions toward healthier habits, these changes were not statistically significant relative to the control group. When viewed together, scores from these prevention strategy implementation scales may indicate that while parents were more likely to make dietary modifications for their children, they were less likely to extend these changes to the entire family. Previous reviews have shown that family-level lifestyle changes often require longer or more intensive interventions (Kurtzhals et al. 2024; Andreae et al. 2024). The design of future genetic counseling interventions for T2D prevention could consider how to incorporate counseling strategies such as motivational interviewing or utilizing partnerships with providers who have regular touchpoints with patients to have a greater impact on behavior changes.

Significant behavior change may have also been limited by the fact that our study consisted of mostly normal weight children whose parents felt they were eating a healthy diet and getting sufficient exercise. Motivation to change habits related to eating and activity may be limited when children are not exhibiting outward signs of risk for T2D, e.g., being overweight. Unfortunately, we did not ask parents what motivated them to implement or not implement prevention strategies to determine if their child’s current health status was a factor.

Although we did not measure motivation, we did measure participant empowerment through the Genomics Outcome Scale (GOS), and our findings suggest that genetic counseling effectively enhanced intervention group participants’ confidence in understanding and utilizing genetic and health-related information. However, GOS scores were not significantly different between the intervention group and control group at 1-month post-intervention. As behavior change is multifaceted, and empowerment is just one factor to consider, future interventions could continue to explore how empowerment effects motivation long-term behavior change.

### Study strengths

This study has several strengths that could be considered in future research or when developing targeted prevention strategies. The use of validated surveys enhances the reliability and validity of the findings. Additionally, the study included a diverse participant population, which strengthens the generalizability of the results to populations at high risk for youth-onset T2D. This study also investigated the effects of the intervention over time, rather than just immediately post-intervention, allowing for evaluation of whether changes in outcomes were sustained; though a one-month follow up period may not have been sufficient to fully incorporate behavior changes related to prevention strategies. The study population was racially diverse, with most participants identifying as Black, which is a key contribution given the disproportionate impact of T2D on racial and ethnic minority groups and the underrepresentation of these populations in prior prevention research (Lawrence et al. 2021). The study also focused on parents of young children, providing insights into prevention at an earlier developmental stage than most previous interventions, which typically target adolescents.

High scores on the GCSS related to the value of the genetic counseling session and appropriate session length indicate the potential practicality of imbedding a brief genetic counseling component into multidisciplinary diabetes care clinics or pediatrician appointments. Incorporating a structured genetic counseling intervention focused on personalized risk and prevention for at-risk families into these already established modalities would provide the opportunity for sustained engagement with families and their existing providers, periodic reinforcement of risk information, and troubleshooting of any prevention efforts that need adjustment over time based on personal and family needs.

### Limitations

Several limitations should be considered when interpreting these findings. One notable limitation is the variability in survey delivery. Surveys were administered in two different modalities, either in person during clinic visits or virtually through a secure online platform. Additionally, participants completed the surveys at different times and locations, which may have introduced variability in responses. Environmental factors such as distractions at home or differences in facilitator engagement between in-person and virtual sessions could have influenced how participants understood and answered survey questions. The surveys were also quite lengthy, which could have introduced survey fatigue. Future studies should aim to standardize survey administration methods to minimize potential biases and ensure consistency in data collection.

The small sample size should also be taken into consideration when extrapolating significant findings, as results may be different in larger populations or populations with different demographic characteristics than our sample. For example, we did not evaluate the effect sizes as part of our statistical analyses, which may have impacted the interpretation of significant results. Importantly, our sample included children who, according to their parents, were overall not overweight, were eating a healthy diet, and were getting sufficient exercise, so they had minimal T2D risk factors other than family history and ethnicity. The intervention may have a different effect on parents whose children have additional risk factors related to weight and low activity levels. While findings provide preliminary support for genetic counseling increasing risk knowledge and behavior related to T2D prevention, larger studies are needed to confirm these results and explore their generalizability to broader populations.

## Conclusion

This study provides evidence that genetic counseling can serve as a tool for educating parents with T2D about their child’s risk of developing T2D and equipping them with knowledge to implement prevention strategies. While the intervention significantly improved risk knowledge and parental monitoring eating behaviors, its effects on overall risk perception and broader family behaviors were less pronounced. These findings highlight the need for further research, specifically for parents whose at-risk children are currently young and healthy. Exploration of how a child’s current age and health status influence risk perception, optimistic bias, and motivation to engage in behavior change across varying age and demographic groups could lead to more personalized T2D education and prevention efforts. Collaboration with diabetes care providers and pediatricians could also optimize genetic counseling interventions for pediatric diabetes prevention efforts. Through further research, refinement, and collaboration, genetic counseling could have a unique role in reducing the incidence of youth-onset T2D and promoting healthier outcomes for at-risk children.

## Supplementary Information

Below is the link to the electronic supplementary material.

ESM 1 (DOCX 35.1 KB)

## Data Availability

De-identified data beyond what is presented in the manuscript is available upon reasonable request sent to the corresponding author.
