# Supplementary material for: The impact of genetic counseling on parental risk perception and prevention strategy utilization for type 2 diabetes in at-risk children
Source: J Community Genet. 2025 Nov 17;17(1):6. doi: 10.1007/s12687-025-00841-z (PMC12627270; doi:10.1007/s12687-025-00841-z)
Supplement: Supplementary file 1 — (DOCX 35.1 KB) [file 12687_2025_841_MOESM1_ESM.docx]

Martina C. Cornel, MD, PhD

Editor-in-Chief

Journal of Community Genetics

September 25, 2025

Dear Dr. Cornel,

I am writing to re-submit our manuscript titled “The Impact of Genetic Counseling on Parental Risk Perception and Utilization of Prevention Strategies for Type 2 Diabetes in At-Risk Children” for consideration as an original research article in the *Journal of Community Genetics*.

In the most recent review, the reviewer recommended two minor revisions, which we feel we have addressed sufficiently in the most recent submission. We look forward to the opportunity to share our findings with the *Journal of Community Genetics* readership.

Please see below for a summary of our responses to the reviewer’s comments and suggestions. Thank you for re-considering the manuscript, and we look forward to hearing back regarding its suitability for the *Journal of Community Genetics.*

Kind regards,

Jessica Denton, PhD, MS, CGC

Licensed and Certified Genetic Counselor; Assistant Professor

University of Alabama at Birmingham

| **Reviewer Comment** | **Author Revisions** |
| --- | --- |
| • In the discussion, paragraph eight, you mention the disproportionate impact of T2D among racial and ethnic minority groups. Please include a literature reference here. I also noticed this was missing in the introduction, where T2D is described as a multifactorial condition (with modifiable and non-modifiable risk factors) and where the inclusion of diverse populations in this study is mentioned, but not explained in terms of why this is important. | Added reference in discussion as requested.  Added information in introduction section to highlight the contributions of race and ethnicity to the risk of T2D in children. We feel this creates a more cohesive connection to why we view our diverse population as a strength as detailed in the discussion. |
| • Since Table 5 has been removed compared to the previous version, the references in the text need to be updated accordingly. All mentions of Tables 6, 7, and 8 should now refer to Tables 5, 6, and 7, respectively. | This has been corrected in the text. |
